# Cognitive and Behavioral Manifestations in ALS: Beyond Motor System Involvement

**DOI:** 10.3390/diagnostics11040624

**Published:** 2021-03-30

**Authors:** Robert Rusina, Rik Vandenberghe, Rose Bruffaerts

**Affiliations:** 1 Department of Neurology, Third Faculty of Medicine, Charles University, and Thomayer University Hospital, 140 59 Prague, Czech Republic; 2 Laboratory for Cognitive Neurology, Department of Neurosciences, Leuven Brain Institute (LBI), KU, 3000 Leuven, Belgium; rik.vandenberghe@uzleuven.be (R.V.); rose.bruffaerts@kuleuven.be (R.B.); 3 Department of Neurology, University Hospitals, 3000 Leuven, Belgium; 4 Biomedical Research Institute, Hasselt University, 3590 Diepenbeek, Belgium

**Keywords:** neurodegeneration, amyotrophic lateral sclerosis, behavioral impairment, frontotemporal lobar degeneration, dementia

## Abstract

Amyotrophic lateral sclerosis (ALS) has long been considered to be a purely motor disorder. However, it has become apparent that many ALS patients develop cognitive and behavioral manifestations similar to frontotemporal dementia and the term amyotrophic lateral sclerosis-frontotemporal spectrum disorder (ALS-FTSD) is now used in these circumstances. This review is intended to be an overview of the cognitive and behavioral manifestations commonly encountered in ALS patients with the goal of improving case-oriented management in clinical practice. We introduce the principal ALS-FTSD subtypes and comment on their principal clinical manifestations, neuroimaging findings, neuropathological and genetic background, and summarize available therapeutic options. Diagnostic criteria for ALS-FTSD create distinct categories based on the type of neuropsychological manifestations, i.e., changes in behavior, impaired social cognition, executive dysfunction, and language or memory impairment. Cognitive impairment is found in up to 65%, while frank dementia affects about 15% of ALS patients. ALS motor and cognitive manifestations can worsen in parallel, becoming more pronounced when bulbar functions (affecting speech, swallowing, and salivation) are involved. Dementia can precede or develop after the appearance of motor symptoms. ALS-FTSD patients have a worse prognosis and shorter survival rates than patients with ALS or frontotemporal dementia alone. Important negative prognostic factors are behavioral and personality changes. From the clinician’s perspective, there are five major distinguishable ALS-FTSD subtypes: ALS with cognitive impairment, ALS with behavioral impairment, ALS with combined cognitive and behavioral impairment, fully developed frontotemporal dementia in combination with ALS, and comorbid ALS and Alzheimer’s disease. Although the most consistent ALS and ALS-FTSD pathology is a disturbance in transactive response DNA binding protein 43 kDa (TDP-43) metabolism, alterations in microtubule-associated tau protein metabolism have also been observed in ALS-FTSD. Early detection and careful monitoring of cognitive deficits in ALS are crucial for patient and caregiver support and enable personalized management of individual patient needs.

## 1. Introduction

Amyotrophic lateral sclerosis (ALS) has long been considered a purely motor disorder, with occasional reports of associated neuropsychiatric symptoms. Since the publication of the seminal case series in 2002, it has become apparent that many ALS patients develop cognitive and behavioral manifestations similar to frontotemporal dementia (FTD) [1] and the term amyotrophic lateral sclerosis-frontotemporal spectrum disorder (ALS-FTSD) is now used in these circumstances.

The recently proposed diagnostic criteria for ALS-FTSD create distinct categories based on the type of neuropsychological manifestation, i.e., changes in behavior, impaired social cognition, executive dysfunction, and language or memory impairment [2]. These manifestations result from a variable degree of frontotemporal dysfunction and atrophy in ALS (Table 1).

Since patients with ALS and dementia also often have transactive response DNA binding protein 43 kDa (TDP-43) positive inclusions, the clinical and neuropathological term frontotemporal lobar degeneration with motor neuron disease and TDP-43 positive inclusions (FTLD-MND-TDP) became widespread, although it is less common today. Dementia is usually clinically defined as a loss of cognitive function that is severe enough to interfere with daily life. However, in this context, terminological inconsistencies with “ALS with dementia” become apparent since for the behavioral form (ALSbi), the criteria for dementia, relative to the loss of autonomy, are often not met. Consequently, the terminology was recently updated from “FTLD-MND” to “ALS-FTSD” (with an emphasis on “spectrum”). The term FTLD-MND-TDP is now used to describe the neuropathological entity, while in clinical practice, different subtypes of ALS-FTSD are still being utilized.

This systematic review is part of a special issue entitled Diagnosis and Treatment of Amyotrophic Lateral Sclerosis: Where We Are and What We Can Expect in the Near Future. Our goal is to present an overview of the cognitive and behavioral manifestations commonly encountered in ALS patients in order to improve case-oriented management in clinical practice. The emerging ALS-FTSD spectrum represents an important group of rare neurodegenerative diseases, and our review aims to raise awareness of the possible phenotypes in non-expert clinicians and hence enable earlier diagnosis. We introduce the principal ALS-FTSD subtypes and comment on their principal clinical manifestations, neuroimaging findings, neuropathological a genetic background, and summarize available therapeutic options.

## 2. Amyotrophic Lateral Sclerosis-Frontotemporal Spectrum Disorder (ALS-FTSD) Subtypes

In 2017, the diagnostic criteria for cognitive and behavioral impairment in ALS (ALSFTD-1) [3] was modified (ALSFTD-2) to include a new category, i.e., ALS with combined cognitive and behavioral impairment, ALScbi, as well as changes to the operational criteria of other categories [2]. The new ALSFTD-2 criteria have a clear clinical impact in that they are more sensitive to early cognitive impairment and anticipate the prognostic aspects [4]. From the clinician’s perspective, ALSFTD-2 has five major distinguishable ALS-FTSD subtypes (Table 1):

1. ALS with cognitive impairment (ALSci);
2. ALS with behavioral impairment (ALSbi);
3. ALS with combined cognitive and behavioral impairment (ALS-cbi);
4. Fully developed behavioral variant of frontotemporal dementia (bvFTD) in combination with ALS (ALS-FTD);
5. Comorbid ALS and Alzheimer’s disease (AD).

ALSci can be screened for by obtaining a letter fluency measure in clinical practice. The formal diagnosis relies on a neuropsychological assessment demonstrating either evident frontal type cognitive deterioration or language impairment [2]. A highly similar cognitive phenotype in the absence of behavioral disturbance can also be observed in primary lateral sclerosis [5].

In current clinical practice, ALSbi can be exceedingly difficult to diagnose because the diagnosis mainly relies on information provided by the proxy. Primary symptoms include major behavior disturbances and personality changes co-occurring with affected social cognition. Apathy is the most frequent behavioral symptom. Patients become irritable and negative, profoundly egocentric, and both their emotional perception and behavior tend to change, which results in dramatically altered social and communication skills (often confirmed by relatives, but only after targeted questioning). However, these manifestations are in stark contrast with the mild degree of cognitive impairment, often assessed using standard neuropsychological assessments of the classic cognitive domains.

ALS-cbi is further characterized by apathy and sometimes severe anosognosia and behavioral disturbances, a neuropsychiatric profile that is very close to fully developed bvFTD. Patients with comorbid ALS and AD typically show a clinical course of progressive amnestic dementia, which is typical for AD, with early and significant impairment of episodic memory with evident hippocampal atrophy on MRI. For more information about useful test batteries, we refer to Section 5 “Cognitive and Behavioral Screening for ALS-FTSD”.

## 3. Prevalence of ALS-FTSD

The prevalence of ALS is 4–8 cases per 100,000 inhabitants in the age group at the highest risk of developing ALS (45–75 years) [6]; cognitive impairment is found in up to 65% of these patients. The degree of cognitive impairment is highly variable: frank dementia affects about 15% of ALS patients [7].

ALS motor and cognitive manifestations can worsen in parallel, becoming more pronounced when bulbar functions (affecting speech, swallowing, and salivation) are involved [8,9]. Manera et al. compared patients according to the lateralization of motor damage and found that spinal patients with symmetric motor impairment had significantly worse cognitive performance than those with lateralized damage [10].

## 4. Clinical Manifestations of ALS-FTSD

The degree of cognitive and behavioral impairment in ALS is variable. Very mild cognitive disturbances, presenting with close to normal performance, are seen in 48.2% of ALS patients. Significant personality and behavioral disturbances, in the context of full-blown FTD, can affect up to 20.5% of ALS patients [8]. A recent study found progressive cognitive and/or behavioral impairment in more than one-third of patients with early symptomatic ALS [11]. The disease timeline is still a matter of debate. Longitudinal studies on the cognitive decline over time in ALS are still missing to the best of our knowledge; however, some cross-sectional studies have suggested poorer cognitive performance in the advanced stages of the disease [12]. It is not clear whether cognitive impairment in ALS (if it occurs) worsens in line with a physical disability.

Dementia can precede or develop after the appearance of motor symptoms. ALS patients, during their illness, may develop a more or less typical profile of bvFTD. In contrast, patients with bvFTD may eventually display signs of motor neuron disease (MND) ranging from subtle lower motor neuron (such as minor fasciculations or mild atrophy without weakness) and upper motor neuron (such as mild hyperreflexia) manifestations without functional impact to typical ALS (confirmed by EMG). In some patients, there is parallel, concomitant development of both the ALS and bvFTD clinical profiles; however, this is unusual [13].

ALS-FTSD patients have a worse prognosis and shorter survival rates than patients with ALS or bvFTD alone. Important negative prognostic factors are behavioral and personality disturbance [2]. When neuropsychological deficits precede the onset of motor involvement, survival is longer than when it is the other way around (median survival of 4.4 years versus 2.7 years) [14]. Early detection and careful monitoring of cognitive deficits in ALS are crucial for patient and caregiver support and enable personalized management of individual patient needs [15].

### 4.1. Executive Dysfunction

One of the first signs of cognitive impairment in ALS-FTSD is decreased letter fluency, which is considered a hallmark of executive dysfunction in this disease. Category verbal fluency is more prominently impaired in patients with co-occurring speech pathology but is not limited to this subgroup [16]. Verbal fluency is also poorer in ALS-FTSD compared to bvFTD, even when controlling for motor impairment [17]. This difference may be linked to more pronounced atrophy of the dorsolateral prefrontal cortex in ALS-FTSD compared to bvFTD [14]. With disease progression, impaired planning, judgment, and task switching or set-shifting appear, and perseveration develops. These manifestations correlate with further involvement of the dorsolateral frontal cortex and the inferior frontal gyrus [18], and their prevalence is comparable to the prevalence of executive dysfunction in bvFTD [19].

### 4.2. Altered Social Cognition & Emotion Processing

Social cognition refers to the processing of social information, and three categories can be distinguished: (1) emotion recognition (ability to identify the emotions of others), (2) empathy (realized and perceived feelings, thoughts, and experience of others), and (3) theory of mind (ability to attribute independent mental states to others in order to understand and predict their behavior).

In FTD syndromes, social cognition may be impaired and should be evaluated [20]. Altered social cognition in ALS-FTSD manifests as a lack of emotional control and perception (for instance, patients may have difficulties recognizing facial emotions) [14,21]. Patients also often manifest a loss of empathy and orientation in complex social situations (for example, they cannot detect a faux pas [22]). These manifestations reflect dysfunction of the orbitofrontal and medial frontal cortex areas.

Uncontrollable emotional outbursts such as pathological laughing and crying are sometimes observed. Contextually appropriate stimuli usually trigger these experiences, but patients cannot voluntarily suppress their emotional responses to these triggers. Pathological laughing and crying is a hallmark of bulbar dysfunction, which is combined with prefrontal dysfunction. The former (laughing) is more frequently context-inappropriate, and the latter (crying) is usually context-appropriate. Dysfunction of the dorsal brain regions regulating emotions (dorsal anterior cingulate, dorsolateral prefrontal cortex) may give rise to this condition [23].

### 4.3. Behavioral Manifestations

Apathy (reduced motivation towards goal-oriented behaviors) is a common manifestation in ALS and can seriously impact caregivers [24,25]. Similarly, in ALS-FTSD, the most frequent behavioral sign is apathy, affecting up to 70% of these patients. Other manifestations include disinhibition, egocentrism, mental rigidity, impulsivity, decreased social adaptation, and loss of empathy and insight (anosognosia) [2]. Apathy occurs as frequently in ALS-FTSD as in bvFTD, whereas the other manifestations are more common in bvFTD compared to ALS-FTSD [19]. In particular, anosognosia is less prevalent in ALS-FTSD compared to bvFTD, leading to more reliable self-reporting of symptoms in patients with ALS-FTSD [17].

### 4.4. Speech and Language Impairment

In ALS-FTSD patients, language impairment is quite common and comparable in severity to that seen in primary progressive aphasia (PPA) [26]. In ALS-FTSD, language symptoms most often occur in the context of a broader frontal lobe syndrome (reflecting the damage to higher functioning processes in the brain such as motivation, planning, social behavior, and language/speech production) rather than an isolated deficit, which is required for a PPA diagnosis [19]. Language impairment is consistently and more frequently observed in ALS-FTD compared to bvFTD [19]. Moreover, patients with ALS-FTSD can present as PPA, either with the language phenotype of the non-fluent/agrammatical variant (nfvPPA) or as the semantic variant (svPPA). In a series of 32 cases with a confirmed neuropathological diagnosis of FTLD-MND, 10 cases presented with prominent or isolated language impairment consistent with a diagnosis of PPA (3 cases resembling nfvPPA and 7 cases resembling svPPA), although both groups displayed subtle motor symptoms at presentation. These consisted of effortful speech, tongue weakness in nfvPPA-MND, and upper limb weakness with fasciculations in both groups. When looking at the timeline, the nfvPPA-MND cases developed severe motor deficits more rapidly than the svPPA-MND cases [9].

Recent evidence regarding language disturbances in ALS-FTSD suggests impaired semantic and syntactic processing. Semantic deficits mostly manifest as anomia during confrontation naming or single word comprehension difficulties. Impaired syntactic processing results in impaired sentence-level comprehension, manifesting as role reversal errors in passive sentences. Although these language problems occur in ALS patients in general, they are more pronounced and more frequent in ALS-FTSD (for instance, syntactic deficits were detected in 25% of ALS patients compared to 92.9% of ALS-FTSD patients). Agrammatism can be as severe in FTD-ALS as in nfvPPA [27]. The importance of semantic deficits in ALS patients tends to be lower than in ALS-FTSD [28]. The degree of atrophy in the anterior temporal areas significantly correlates with semantic deficits, whereas left peri-insular atrophy corresponds to impaired syntactic comprehension [27].

A loss of communication skills in ALS-FTSD patients, especially those related to speech impediments, can have a significant impact. In the early stages of ALS, the type of dysarthria can be either flaccid or spastic, but with disease progression, the typical flaccid-spastic mixed dysarthria is more frequently observed. ALS lesions can be located in the primary motor cortex and/or descending corticobulbar tracts (upper motor neurons and/or their axons), cranial nerve motor nuclei in the pons, and medulla oblongata (the lower motor neurons and their axons). Changes in speech production may be influenced by several factors; for example, reduced speech rates may result from dysarthria and difficult word retrieval from the patient’s mental lexicon. Automated speech analysis demonstrated that prosodic variations (measured using the fundamental frequency, f0, i.e., the physical property of sound most closely correlating with perceived pitch) were pathologically reduced in ALS-FTD compared to controls. These changes were associated with atrophy of the motor cortex and the degree of bulbar involvement, although other mechanisms such as impaired processing of emotions may also play a role [29].

### 4.5. Memory Impairment

In ALS-FTSD, episodic memory (the ability to recall the temporal and spatial context of past experiences) is relatively preserved, even though attention deficits can influence overall performance. These results are supported by studies using animal models. A recent study detected early cognitive alteration and damage to the γ-aminobutyric acid (GABAergic) system in the hippocampus using an ALS murine model, suggesting selective deficits that are antecedent to the onset of motor symptoms [30].

Potential memory deficits would have more impact on retrieval than on storage of recent memory traces. In a recent study, ALS patients were shown to have substantial deficits in verbal learning, immediate and delayed recall, and recognition compared to healthy controls [31].

Episodic memory deficits can occur in ALS-FTSD patients; most commonly, if there is a memory deficit, it remains relatively stable and may reflect early impairment of cognition in the disease and may correlate with involvement of temporal lobe structures (i.e., hippocampus, entorhinal, and parahippocampal cortices) [31].

In ALS-FTSD cases with early impairment in episodic memory, incidental co-occurrence of Alzheimer’s disease should be considered. If this is the case, neuropsychological testing will then reveal a classic hippocampal memory profile, with a decrease in both encoding and retrieval, impaired learning abilities, and numerous intrusions. The same consideration should be made when ALS-FTSD is combined with language impairments fulfilling the criteria for the logopenic variant of PPA (lvPPA), with disproportionate word retrieval difficulties. Analysis of the cerebrospinal fluid for levels of beta-amyloid (1–42), phosphorylated tau, and total tau can help establish a diagnosis of ALS-dementia.

## 5. Cognitive and Behavioral Screening for ALS-FTSD

Cognitive assessment in ALS is often difficult in terms of realization and interpretation because of the sometimes very pronounced dysarthria and motor weakness, and these limitations should be considered when using a particular test battery. Some tests of executive function are timed, e.g., the block design (the participant needs to manipulate small cubes to reconstruct a pattern) and the trail-making test. In ALS-FTSD, impaired motor abilities, as well as executive dysfunction, can contribute to slow performance and hence lower scores. This should be taken into account when interpreting the test results and an untimed version may provide more interpretable results under such conditions.

Screening assessments should focus mainly on verbal fluency testing. For a more detailed evaluation, the ECAS scale (Edinburgh Cognitive and Behavioral ALS Screen) may be helpful [32]. Interestingly, cognitive impairment detected by ECAS seems to be a valid predictor of TDP-43 pathology in non-demented ALS [33].

Another ALS-dedicated screening tool is the Amyotrophic Lateral Sclerosis Cognitive Behavioral Screen (ALS-CBS), which is composed of two components, i.e., the cognitive and behavioral subscales. Woolley et al. suggested using the ALS-CBS to detect cognitive and behavioral impairment in a clinical setting, although it does not replace formal diagnostic assessments [34].

An essential part of the assessment should include a structured interview with the caregiver focused on recent changes in the patient’s cognition and behavior. Whereas ALS-CBS and ECAS are the most suitable screening tools for cognitive changes, the Beaumont Behavioral Inventory (BBI) was proposed as the best screen for behavioral disturbance in a recent review. The BBI summarizes the patient’s family member/caregiver’s evaluation of all relevant domains (disinhibition, apathy, empathy, perseverations, hyperorality) while taking into account motor problems [35].

## 6. Neuroimaging

In contrast to the frequent and marked atrophy (with asymmetry) in bvFTD, ALS-FTSD patients may have a normal MRI. Atrophy, if present, typically prevails in the frontal areas spreading to the temporal lobes and is best seen on coronal slices [36].

A recent study demonstrated that distinct patterns of focal cortical atrophy exist and can differentiate non-motor clinical profiles in ALS patients; this is in line with the concept of FTSD and suggests that inferior frontal, temporal, cingulate, and insular thinning can be used as markers for cognitive and behavioral deficits, with language impairment being mainly associated with the left temporal pole and the insula [28,37]. Strikingly, atrophy of the motor cortex is more pronounced in ALS-FTSD compared to ALS [38,39] and implies a poorer prognosis [14].

Cortical thinning is presumably one of the first structural changes in ALS-FTD and correlates well with hypometabolism on an FDG-PET [40]. When comparing FTSD-ALS variants, moderate frontal hypometabolism was observed in ALSci and ALScbi compared to pure ALS, whereas extensive frontal hypometabolism was found in FTD-ALS. In contrast, no metabolic changes were found in ALSbi compared to ALS [41].

In the orbitofrontal, cingulate, and opercular white matter tracts, degeneration is consistently observed in FTD-ALS compared to ALS [39]. White matter changes in the corticospinal tracts in FTSD-ALS were less prominent compared to ALS but more evident than the changes observed in bvFTD [42]. Changes within the frontostriatal and frontoparietal networks, based on resting-state functional connectivity (rs-FC) in ALS patients, progress over time. Resting-state changes are related to frontal-executive dysfunction and represent new potential markers for monitoring extra-motor progression in ALS [43].

Neuroimaging can help raise suspicion of a genetic form of ALS even in the absence of a family history. Atrophy of the thalamus may be found in *C9orf72* repeat expansions, focal mesial temporal atrophy in *MAPT* mutations, parietal lobe atrophy was reported in *C9orf72, GRN,* and *TBK1* carriers, and prominent cervical spinal cord atrophy in cases with *SOD1* variants [44].

## 7. Genetic Background

The most frequent causal mutation associated with hereditary ALS (60–70%) and a common mutation seen in genetic bvFTD (18%) is a hexa-repeat (GGGGCC) expansion in the *C9orf72* gene. Carriers of the *C9orf72* expansion are at considerably increased risk of cognitive impairment (40–50%) compared to patients without this mutation (8–9%) [8,45]. The typical clinical profile of ALS patients who should be suspected of carrying this expansion includes psychotic features and major anosognosia. The *C9orf72* repeat expansion has also been associated with language impairment [17].

More than 20 other genetic modifications associated with ALS-FTSD have been recognized: from 2% to 6% of these patients have at least one modification. Pathogenic mutations have also been reported in genes for the following proteins: TDP-43 (*TARDBP*), progranulin (*PGRN*), superoxide dismutase 1 (*SOD1*), fused in sarcoma protein (*FUS*), the isoform 5A of heavy chain in kinesin (*KIF5A*), coiled-coil-helix-coiled-coil-helix domain containing 10 (*CHCHD10*), dynactin 1 (*DCNT1*), matrin 3 (*MTR3*), optineurin (*OPTN*), sequestosome 1 (*SQSTM1*), TANK binding kinase 1 (TBK1), tubulin alpha 4A (TUBA4A), ubiquilin-2 (*UBQLN2*), and the valosin containing peptide (*VCP*) [46,47]. Due to the founder effect, large regional differences exist in the frequencies of these mutations [45].

There is a surprising overlap between ALS and bvFTD in terms of genetic background since the same genes can cause ALS and FTD (i.e., mutations in *C9orf72*, *TARDBP*, *FUS*, *TBK1*, *VCP*, *CHCHD10*, *TIA1*, and *SQSTM1*). This overlap, however, is not complete: *SOD1*, *FUS*, and TDP-43 variants are often found in ALS but rarely in bvFTD patients. By contrast, *GRN* and *MAPT* are strongly linked to FTD, but rarely (in about 1% of mutation carriers) cause ALS-FTSD [45,48].

The benefit of routine genetic testing for ALS-FTSD patients is still a matter of discussion; however, it should be recommended if there is a family history, an atypical clinical course (for instance, PPA-ALS [49]), or atypical neuropathology findings on autopsy [50].

## 8. Neuropathology

Key micromorphological findings include different types of diagnostic inclusions that have positive immunohistochemical reactions with antibodies against ubiquitin and protein p62, which is typically found in motor neurons of the anterior spinal horns, the primary motor cortex, and neuronal structures in the frontal and temporal cortex. Most neuronal structures contain cytoplasmic inclusions that have positive reactions with anti-TDP-43 antibodies, mainly the hyperphosphorylated form. These structures are identical to those found in ALS without cognitive impairment [51]. In genetic ALS-FTSD, other inclusions of abnormally modified proteins can be found, most frequently SOD1 and FUS, although rare.

Animal studies support the importance of TDP-43 and FUS in ALS pathogenic mechanisms. Suppression of human TDP-43(A315T) expression in mice with overt neurodegeneration for only one week was sufficient to significantly improve motor and behavioral deficits and reduce astrogliosis [52]. In an experimental study, FUS transgenic rats reproduced certain phenotypes of ALS and FTLD [53].

Although the most consistent pathology of ALS and ALS-FTSD is a disturbance in TDP-43 metabolism, alterations in microtubule-associated tau protein (tau) metabolism have also been observed in ALS-FTSD [54]. The PPA-ALS phenotype is most consistently associated with FTLD-TDP-MND [9,49].

## 9. Therapeutic Options

Currently available therapeutic options for ALS-FTSD are based on the treatment of the MND and bvFTD components and include both pharmacological and non-pharmacological interventions.

For MND, therapy includes riluzole, supportive treatment, physiotherapy, speech therapy, percutaneous endoscopic gastrostomy (PEG), and non-invasive ventilation in selected cases. Recently, edaravone was approved in selected countries [6]. Treatment of the bvFTD component may consist of serotonergic agents, antipsychotic drugs, and memantine. In ALS-AD comorbidity, acetylcholinesterase inhibitors are prescribed.

As with the management of bvFTD and ALS patients, cooperation with relatives and support for caregivers is very important. An early discussion regarding the prognosis is important because of the anticipated decline over time. A clear understanding of therapeutic prospects is crucial for both the patient and their family [55]. The patient’s decisions regarding their care should be expressed in the form of an advance directive and put into writing while the patient is still competent to make such decisions [56].

## 10. Conclusions

The emerging ALS-FTSD spectrum represents a heterogeneous group of rare neurological diseases. Increased awareness of these complex phenotypes is needed to enable earlier diagnosis and optimal patient care. Our review is part of concerted action by the European Reference Network for Rare Neurological Diseases to promote the transfer of expert knowledge to non-expert clinicians in the field and responds to an unmet educational need regarding clinical manifestations of rare neurodegenerative disorders such as the ALS-FTSD spectrum [57]. The field of ALS-FTSD is rapidly evolving, and we anticipate that synthesizing and sharing current state-of-the-art knowledge will trigger new clinical insights and foster international collaboration. This brings us closer to our goal of providing optimal quality of care to patients with rare neurodegenerative diseases around the world.

## Figures and Tables

**Table 1:** Amyotrophic lateral sclerosis-frontotemporal spectrum disorder (ALS-FTSD) criteria for the diagnosis of cognitive and behavioral symptoms associated with ALS, modified following Strong et al. [2].

ALS-FTSD Variant	Acronym	Definition
ALS with behavioral impairment	ALSbi	Apathy with or without other behavioral changes or Presence of two or more behavioral/cognitive changes associated with bvFTD *
ALS with cognitive impairment	ALSci	Evidence of either executive dysfunction/social cognition or language dysfunction or a combination of the two Executive impairment criteria: impaired letter fluency OR impairment on 2 non-overlapping tests of executive dysfunction (may include social cognition) Language impairment: impairment on 2 non-overlapping tests in which language impairment is not explained by a decrease in verbal fluency
ALS with combined cognitive and behavioral impairment	ALS-cbi	Criteria fulfilled for both ALSci and ALSbi
ALS with frontotemporal dementia	ALS-FTD	Progressive deterioration of behavior and/or cognition (observation or history) and Presence of at least three behavioral/cognitive changes associated with bvFTD * or At least two behavioral/cognitive changes associated with bvFTD * together with loss of insight and/or psychotic symptoms or Fulfilled criteria for primary progressive aphasia (non-fluent/agrammatical variant or semantic variant)
ALS with comorbid dementia	ALS-dementia	Comorbid ALS and Alzheimer’s disease/other dementia than FTD Mixed dementia (ALS and vascular dementia)
FTLD-MND-like		Neuropathological evidence of FTLD and neuropathological hallmarks of ALS but the clinical diagnostic criteria of ALS are not met

* Behavioral/cognitive manifestations of bvFTD: Early behavioral disinhibition; Early apathy; Early loss of sympathy and empathy; Early perseverative, stereotyped, or compulsive behavior; Hyperorality/dietary change; Neuropsychological profile: executive deficits with relative sparing of memory and visuospatial functions. For abbreviations, see the list at the end of this article.

## Abbreviations

AD	Alzheimer’s disease
ALS	Amyotrophic Lateral Sclerosis
ALSbi	ALS with behavioral impairment
ALSci	ALS with cognitive impairment
ALScbi	ALS with combined cognitive and behavioral impairment
ALS-FTD	fully developed bvFTD in combination with ALS
ALSFTD-2	modified diagnostic criteria for cognitive and behavioral impairment in ALS
ALS-FTSD	Amyotrophic Lateral Sclerosis–Frontotemporal Spectrum Disorder
bvFTD	behavioral variant of frontotemporal dementia
FTLD	frontotemporal lobar degeneration
FTLD-MND	frontotemporal lobar degeneration with motor neuron disease
MND	motor neuron disease
nfvPPA	non-fluent/agrammatical variant of primary progressive aphasia
svPPA	semantic variant of primary progressive aphasia
TDP-43	transactive response DNA binding protein 43 kDa
